# Missing value imputation for microarray data: a comprehensive comparison study and a web tool

**DOI:** 10.1186/1752-0509-7-S6-S12

**Published:** 2013-12-13

**Authors:** Chia-Chun Chiu, Shih-Yao Chan, Chung-Ching Wang, Wei-Sheng Wu

**Affiliations:** 1 Department of Electrical Engineering, National Cheng Kung University, No.1 University Road, 701 Tainan, Taiwan (R. O. C

**Keywords:** microarray, missing value, imputation, evaluation

## Abstract

**Background:**

Microarray data are usually peppered with missing values due to various reasons. However, most of the downstream analyses for microarray data require complete datasets. Therefore, accurate algorithms for missing value estimation are needed for improving the performance of microarray data analyses. Although many algorithms have been developed, there are many debates on the selection of the optimal algorithm. The studies about the performance comparison of different algorithms are still incomprehensive, especially in the number of benchmark datasets used, the number of algorithms compared, the rounds of simulation conducted, and the performance measures used.

**Results:**

In this paper, we performed a comprehensive comparison by using (I) thirteen datasets, (II) nine algorithms, (III) 110 independent runs of simulation, and (IV) three types of measures to evaluate the performance of each imputation algorithm fairly. First, the effects of different types of microarray datasets on the performance of each imputation algorithm were evaluated. Second, we discussed whether the datasets from different species have different impact on the performance of different algorithms. To assess the performance of each algorithm fairly, all evaluations were performed using three types of measures. Our results indicate that the performance of an imputation algorithm mainly depends on the type of a dataset but not on the species where the samples come from. In addition to the statistical measure, two other measures with biological meanings are useful to reflect the impact of missing value imputation on the downstream data analyses. Our study suggests that local-least-squares-based methods are good choices to handle missing values for most of the microarray datasets.

**Conclusions:**

In this work, we carried out a comprehensive comparison of the algorithms for microarray missing value imputation. Based on such a comprehensive comparison, researchers could choose the optimal algorithm for their datasets easily. Moreover, new imputation algorithms could be compared with the existing algorithms using this comparison strategy as a standard protocol. In addition, to assist researchers in dealing with missing values easily, we built a web-based and easy-to-use imputation tool, MissVIA (http://cosbi.ee.ncku.edu.tw/MissVIA), which supports many imputation algorithms. Once users upload a real microarray dataset and choose the imputation algorithms, MissVIA will determine the optimal algorithm for the users' data through a series of simulations, and then the imputed results can be downloaded for the downstream data analyses.

## Background

Gene expression microarray (DNA chip) technology is a powerful tool for modern biomedical research. It could monitor relative expression of thousands of genes under a variety of experimental conditions. Therefore, it has been used widely in numerous studies over a broad range of biological disciplines, such as cell cycle regulation, stress responses, cancer diagnosis, functional gene discovery, specific therapy, and drug dynamic identification [1-9]. Although microarray technology has been used for several years, expression data still contain missing values due to various reasons such as scratches on the slide, spotting problems, poor hybridization, inadequate resolution, fabrication errors and so on.

Basically, microarray data contain 1-10% missing values that could affect up to 95% of genes [10]. The occurrence of missing values in microarray data disadvantageously influences downstream analyses, such as discovery of differentially expressed genes [11,12], construction of gene regulatory networks [13,14], supervised classification of clinical samples [15], gene cluster analysis [10,16], and biomarker detection.

One straightforward solution to solve the missing value problem is to repeat the microarray experiments, but that is very costly and inefficient. Another solution is to remove genes (rows) with one or more missing values before downstream analysis, but it is easily seen that part of important information would be lost. Hence, advanced algorithms must be developed to accurately impute the missing values.

Using modern mathematical and computational techniques can effectively impute missing values. Early approaches included replacing missing values by zero, row average or row median [17]. Recently, many studies found that merging information from various biological data can significantly improve the missing values estimation. Liew *et al. *categorized the existing algorithms into four different classes: (1) local algorithms, (2) global algorithms, (3) hybrid algorithms, and (4) knowledge assisted algorithms [18,19].

The first category includes *k *nearest neighbors (KNN) [17], iterative *k *nearest neighbors (IKNN) [20], sequential *k *nearest neighbors (SKNN) [21], least squares adaptive (LSA) [22], local least squares (LLS) [23], iterative local-least-squares (ILLS) [24], sequential local-least-squares (SLLS) [25], and etc. The second category includes *Bayesian *principal component analysis (BPCA) [26], singular value decomposition (SVD) [17], partial least squares (PLS) and so on. The third category includes LinCmb [11]. The fourth category integrates domain knowledge (Gene Ontology [27] and multiple external datasets [18]) or external information into the imputation process. Projection onto convex sets (POCS) [28], GOimpute, histone acetylation information aided imputation (HAIimpute) [29], weighted nearest neighbors imputation (WeNNI) [30] and integrative missing value estimation (iMISS) [31] belong to the knowledge assisted approach algorithms. In this study, we did not use the hybrid algorithms and the knowledge assisted algorithms because their programs are not freely available or cannot be easily modified.

In the past few years, several papers have preliminary and objective analyses for the systematic evaluation of different imputation algorithms [32-35]. The weaknesses of these studies are as follows. First, few microarray datasets were used [32]. Second, few independent rounds of the imputed procedure were performed (usually 10 times). Third, single performance measure was used [33,34]. Here, we present a fair and comprehensive evaluation to assess the performances of different imputation algorithms on different datasets using different performance measures.

## Methods

### Datasets

Considering that datasets from different species and types of datasets may have different effects on the performance of imputation algorithms, we chose thirteen different datasets from two species (*Saccharomyces cerevisiae *and *Homo sapiens*), which could be categorized into three different types (time series, non-time series and mixed type), for our analyses.

For time series datasets, we selected the yeast cell cycle data (including the alpha factor arrest and elutriation datasets) from [36], and Shapira04A and Shapira04B datasets, which were two different time series datasets (both measured the effect of oxidative stress on the yeast cell cycle) from [37]. We also chose the human cell cycle data called Human HeLa from [38]. For non-time series datasets, we chose the datasets (Ogawa, BohenSH and BohenLC) from [39] and [40]. Ogawa's data was retrieved from the study of phosphophate accumulation and poly-phosphophate metabolism and the BohenSH was retrieved from follicular lymphoma lymph node and normal lymph node and spleen samples on SH microarrays and the BohenLC was retrieved from 24 independent follicular lymphoma lymph node samples on LC microarrays. For mixed type datasets, we chose the datasets from Lymphoma [41] (focused on two experimental subsets corresponding to Blood B cells and Thymic T cells), Baldwin [42], Yoshimoto02 [43], Brauer05 [44] and Ronen05 [45].

Before analyses, we removed all genes with missing values to create complete matrices. And then multiple entries with different missing rates (1%, 5%, 10%, 15% and 20%) were randomly introduced into these complete matrices. A brief information of these datasets is presented in Table 1.

**Table 1:** Benchmark datasets.

			Datasets	
**Name**	**Full Dim.**	**Used Dim.**	**Category**	**Species**

Ogawa	6263*8	3069*8	Non-time series	*S.cerevisiae*
Brauer05	6133*60	706*60	Mixed type	*S.cerevisiae*
Ronen05	6987*26	2998*26	Mixed type	*S.cerevisiae*
Yoshimoto02	6166*24	4380*24	Mixed type	*S.cerevisiae*
Spahira04A	4771*23	2970*23	Time series	*S.cerevisiae*
Spahira04B	4771*14	3340*14	Time series	*S.cerevisiae*
Spellman ELU	6178*14	5766*14	Time series	*S.cerevisiae*
Spellman AFA	6178*18	4489*18	Time series	*S.cerevisiae*
BohenSH	2364*24	623*24	Non-time series	*H.sapiens*
BohenLC	13121*24	615*24	Non-time series	*H.sapiens*
Lymphoma	4026*16	2209*16	Mixed type	*H.sapiens*
Baldwin	16838*39	6850*39	Mixed type	*H.sapiens*
Human HeLa	1134*19	920*19	Time series	*H.sapiens*

### Collection of missing value imputation algorithms

In this paper, we present a comprehensive evaluation on the performance of nine imputation algorithms on a wide variety of types and sizes of microarray datasets. We assessed the performance of different algorithms on each dataset. Algorithms used can be divided into two categories: local imputation algorithms and global imputation algorithms.

Local imputation algorithms select a group of genes with the highest relevance (using Euclidian distance [17,23], *Pearson *correlation [22,23], or covariance estimate [46]) to the target gene to impute missing values. For local imputation algorithms, we used k-Nearest-Neighbors (KNN), iterative k-Nearest-Neighbors (IKNN), sequential k-Nearest-Neighbors (SKNN), least squares adaptive (LSA), local least squares (LLS), iterative LLS (ILLS) and sequential LLS (SLLS). For global imputation algorithms, we used singular value decomposition (SVDimpute) and Bayesian principal components analysis (BPCA). The KNN and SVD algorithms were run with the parameter *k *= 15, the SKNN algorithm was run with the parameter *k *= 10 for time series data and *k *= 15 for non-time series data. The automatic parameter estimator was used for LLS, SLLS and BPCA. The LS, IKNN and ILLS methods do not contain any free parameters. A brief information of these algorithms being used is presented in Table 2.

**Table 2:** Missing value imputation methods used in this study

Methods	Author	Programming Language	Year
**Local algorithm**			

K-nearest neighbors (KNN)	Troyanskaya O.	C	2001
Iterative K-nearest-neighbors (IKNN)	Bras L.P.	R	2007
Sequential K-nearest-neighbors (SKNN)	Kim K.Y.	R	2004
Least squares adaptive (LSA)	Bø T.H.	Java	2004
Local least squares (LLS)	Kim H.	Matlab	2005
Iterative local least squares (ILLS)	Cai Z.	Matlab	2006
Sequential local least squares (SLLS)	Zhang X	R	2008

**Global algorithm**			

Bayesian principal component analysis (BPCA)	Oba S.	R	2003
Singular value decomposition (SVD)	Troyanskaya O.	R	2001

### Performance indices

We used three performance indices (normalized root mean squared error, cluster pair proportions and biomarker list concordance index) to assess the performance of imputation algorithms. Based on the type of information used in the index, we categorized these three indices into three different types: (i) statistic index, (ii) clustering index and (iii) differentially expressed genes index.

#### (i) Statistic index

For the statistic index, we used the normalized root mean squared error (NRMSE) to evaluate the performance of the imputation algorithms. Lower the value of the statistic index, better the algorithm performs.

*Normalized root mean squared error (NRMSE): *NRMSE is a popular index used to evaluate the similarity between the true values and the imputed values [33].

(1) $$NRMSE=\sqrt{\frac{mean\left[{\left(y_{guess}-y_{answer}\right)}^{2}\right]}{variance\left[y_{answer}\right]}}$$

where **y_guess _**and **y_answer _**are vectors, the elements of **y_guess _**are the imputed values, the elements of **y_answer _**are the known answer values, and variance[**y_answer_**] is the variance of **y_answer_**.

#### (ii) Clustering index

An important data analysis in the microarray data is the gene clustering. In this study, *k*-means was used to do gene clustering for the complete datasets and the imputed datasets. We used cluster pair proportions (CPP) [10] as a clustering index to evaluate the performance of the algorithms. The numbers of clusters for each dataset was 10. Higher the value of the clustering index, better the algorithm performs.

**Figure 1:**
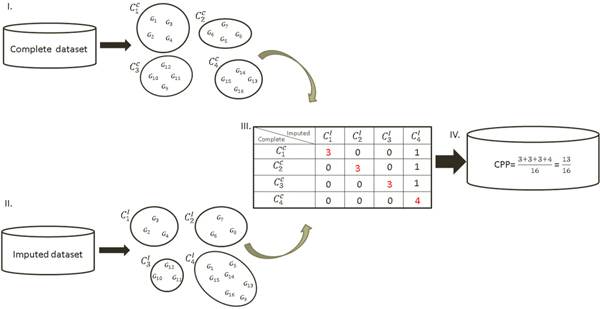
**CPP**. *G*_1_,*G*_2_, ..., *G*_16 _are genes in the microarray data. $C_{1}^{C},\ldots ,C_{4}^{C}$ are the four clusters for the complete dataset. $C_{1}^{I},\ldots ,C_{4}^{I}$ are the four clusters for the imputed dataset.

*Cluster Pair Proportions (CPP): *A schematic illustration of CPP is showed in Figure 1.

#### (iii) Differentially expressed genes index

An important data analysis in the microarray is the identification of differentially expressed genes. In this study, SAM was used to identify differentially expressed genes for the complete dataset and the imputed dataset. We used biomarker list concordance index (BLCI) [47] as the differentially expressed genes index to evaluate the performance of the algorithms.

*Biomarker list concordance index (BLCI): *A high BLCI value indicates that the list of the significantly differentially expressed genes of the complete data is similar to that of the imputed data. And it also means that the imputed data does not significantly change the result of downstream analysis, so the algorithm has excellent performance. We expect that a good algorithm has a high BLCI value. The BLCI is defined as follows:

(2) $$BLCI\left(B_{CD},B_{ID}\right)=\frac{n\left(B_{CD}\cap B_{ID}\right)}{n\left(B_{CD}\right)}+\frac{n\left(B_{CD}^{C}\cap B_{ID}^{C}\right)}{n\left(B_{CD}^{C}\right)}-1,$$

where *B_CD _*is the significantly differentially expressed genes from the complete data, and *B_ID _*is the significantly differentially expressed genes from the imputed data. $B_{CD}^{C}$ is the complement set of *B_C D_*, and $B_{ID}^{C}$ is the complement set of *B_ID_*.

## Results and discussion

We used (i) thirteen different datasets coming from two organisms (human and yeast), (ii) 110 independent rounds per experiment, and (iii) three kinds of indices to assess nine different algorithms. We thought that the performances of algorithms should be evaluated using measures which can reflect the impact of imputation on downstream analysis. The cluster pair proportions (CPP) is used to assess the results of clustering analysis and the biomarker list concordance index (BLCI) is used to assess the results of identifying differentially expressed genes. Therefore, we used not only normalized root mean squared error (NRMSE), but also CPP and BLCI to evaluate the performance of each algorithm. Such a comprehensive comparison can provide an explicit direction for practitioners and researchers for advanced studies.

### Simulation setting

In our numerical experiments, thirteen real microarray datasets were used as benchmark datasets and nine algorithms including KNN, SKNN, IKNN, LS, LLS, ILLS, SLLS, BPCA and SVD were used.

**Figure 2:**
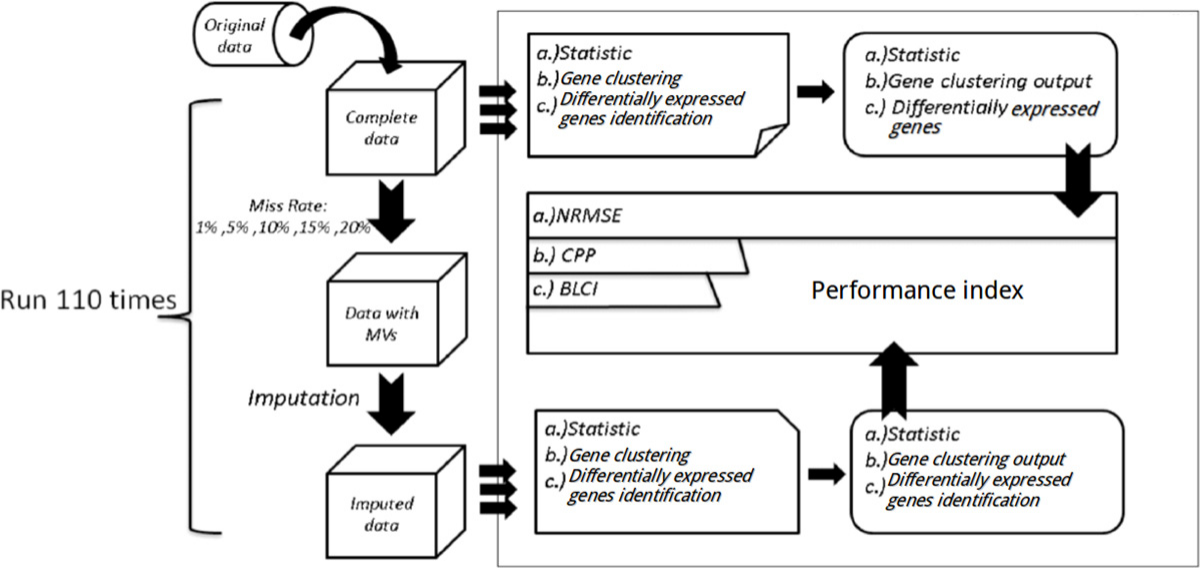
**The diagram of the experiment design**. (a) is the evaluation using the statistic measure to compare the degree of difference between the complete entries and the imputed entries. (b) and (c) are evaluations using indices with biological meanings to compare the impact of imputation on downstream analysis.

First, we removed genes with one or more missing values from the original datasets to generate complete data matrices. Second, multiple entries with different missing percentages (1%, 5%, 10%, 15% and 20%) were randomly introduced into these complete data matrices. And then, the data with missing values was imputed by nine algorithms, respectively. The three steps mentioned above are repeated 110 times for each algorithm. Finally, downstream analysis results from the complete data are compared to the results from the imputed data using three kinds of indices. The workflow of numerical experiments is shown in Figure 2.

### The performances of imputation algorithms

**Figure 3:**
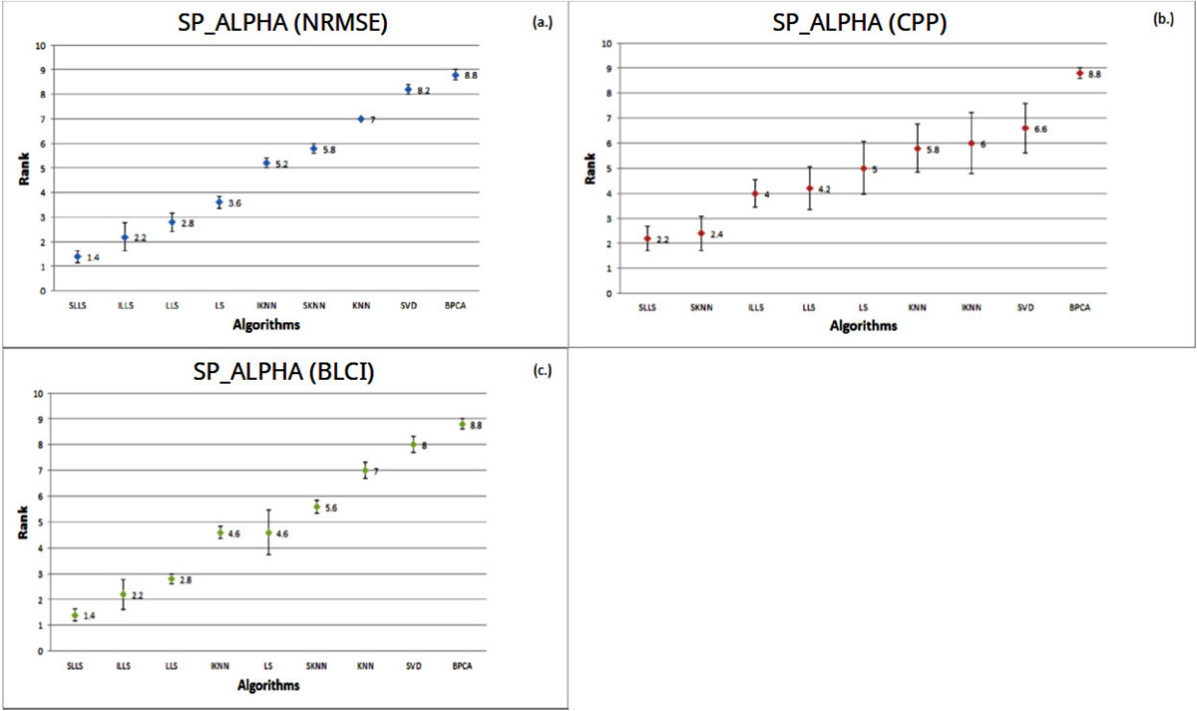
**Performance comparison of different methods on SP_ALPHA**. In the SP_ALPHA, the performances of all algorithms were estimated by three indices (NRMSE, CPP and BLCI). Each point represents the average rank for each algorithm. Different colors (blue, red and green) represent the results evaluated by different indices. The error bar is the standard error.

We present a distinct illustration that can point out the optimal method for the microarray datasets used. The x-axis means the algorithms used and the y-axis means the average rank of each algorithm. For example, if we perform an experiment with 5 independent rounds, in which ranks of an algorithm are 1, 2, 2, 1 and 2 respectively. The average rank of the algorithm in this experiment is (1 + 2 + 2 + 1 + 2)/5 = 1.6. Thus, in Figure 3a, the average rank of SLLS is 1.4, which is the result from 110 rounds in an experiment. The error bar for each algorithm is the standard error of the rank.

In this paper, we compared the performances of imputation algorithms using microarrays of various data types to determine the optimal algorithm. Time series, non-time series and mixed type datasets were used as benchmark datasets, and the performance of each algorithm was evaluated using different measures mentioned above. Furthermore, robustness of an imputation algorithm was also disscussed. We compared robustness of an algorithm between various conditions, such as types of datasets and datasets from samples of different organisms.

### The ranking of imputation algorithms for different data types

#### Performance of imputation algorithms on time series data

**Figure 4:**
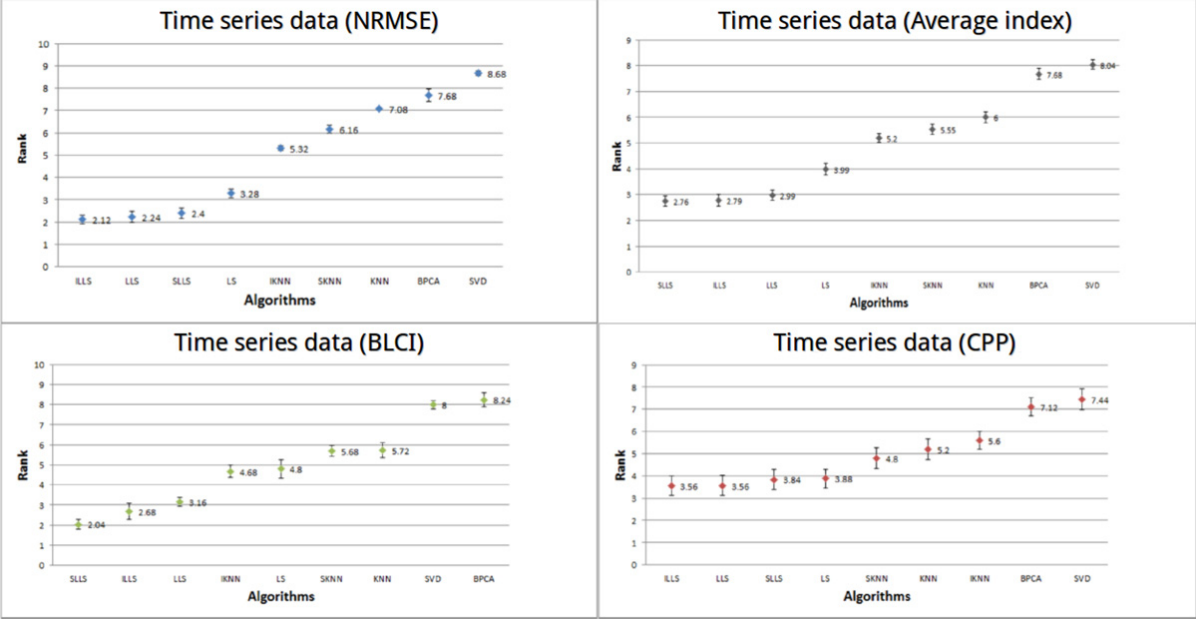
**Performances of different methods on time-series datasets**. In the time series datasets, the performances of all algorithms were estimated by three indices (NRMSE, CPP and BLCI) and the average index. Each point represents the average rank for each algorithm. Different colors (blue, red, green and gray) represent the results evaluated by different indices. The error bar is the standard error.

In Figure 4, LLS-like algorithms (based on local least squares methods, such as LLS [23], ILLS [24] and SLLS [25]) outperform the others on NRMSE. ILLS is the algorithm with the best performance among the LLS-like algorithms (the average rank = 2.12). The average rank of LS and LLS-like algorithms are around 3.8 using the CPP. SLLS is the optimal method using BLCI (average rank = 2.04).

The performances (average rank) of algorithms are estimated by different indices. The optimal algorithm is ILLS using NRMSE (average rank = 2.12), the optimal algorithms are ILLS and LLS using CPP (average rank = 3.56) and the optimal algorithm is SLLS using BLCI (average rank = 2.04). To precisely understand the performances of the algorithms on time series datasets, we averaged each average rank of the algorithms using the different indices as the average rank of the algorithms using the average index on time series datasets. The performance of LLS-like algorithms perform well using the average index. The top two of LLS-like algorithms are SLLS and ILLS. The average rank of SLLS is 2.76 and the average rank of ILLS is 2.79.

#### Performance of imputation algorithms on non-time series data

**Figure 5:**
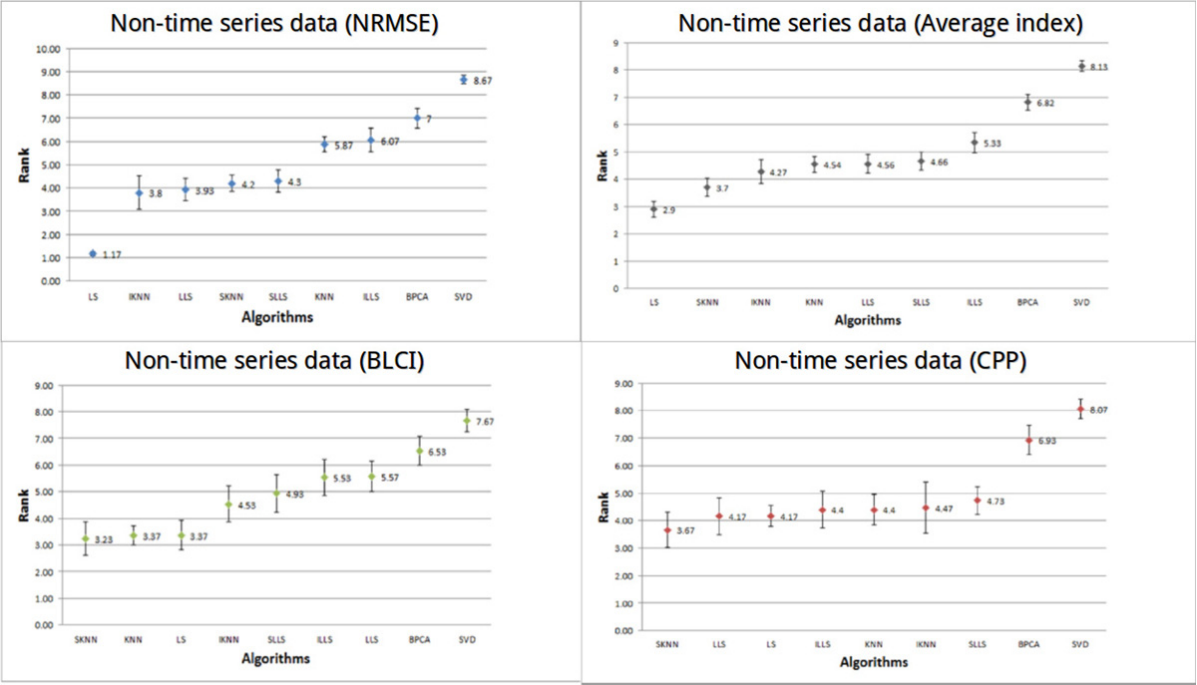
**Performances of different methods on non-time series datasets**. In the non-time series datasets, the performances of all algorithms were estimated by three indices (NRMSE, CPP and BLCI) and the average index. Each point represents the average rank for each algorithm. Different colors (blue, red, green and gray) represent the results evaluated by different indices. The error bar is the standard error.

For non-time series datasets (Figure 5), it is prominent that the performance of LS is the best using NRMSE. The average rank of LS is 1.17. Using BLCI, the three algorithms (SKNN, KNN and LS) have the best performance. The average rank of SKNN is 3.23, the average rank of KNN is 3.37 and the average rank of LS is 3.37. The top performing algorithm is SKNN using CPP. The average rank of SKNN is 3.67. In Figure 5, LS is the optimal algorithm using the average index and then is KNN-based algorithms, such as KNN [17], IKNN [20] and SKNN [21]. We can clearly see that LLS-like algorithms have better performance on time series datasets than on the non-time series datasets.

#### Performance of imputation algorithms on mixed type data

**Figure 6:**
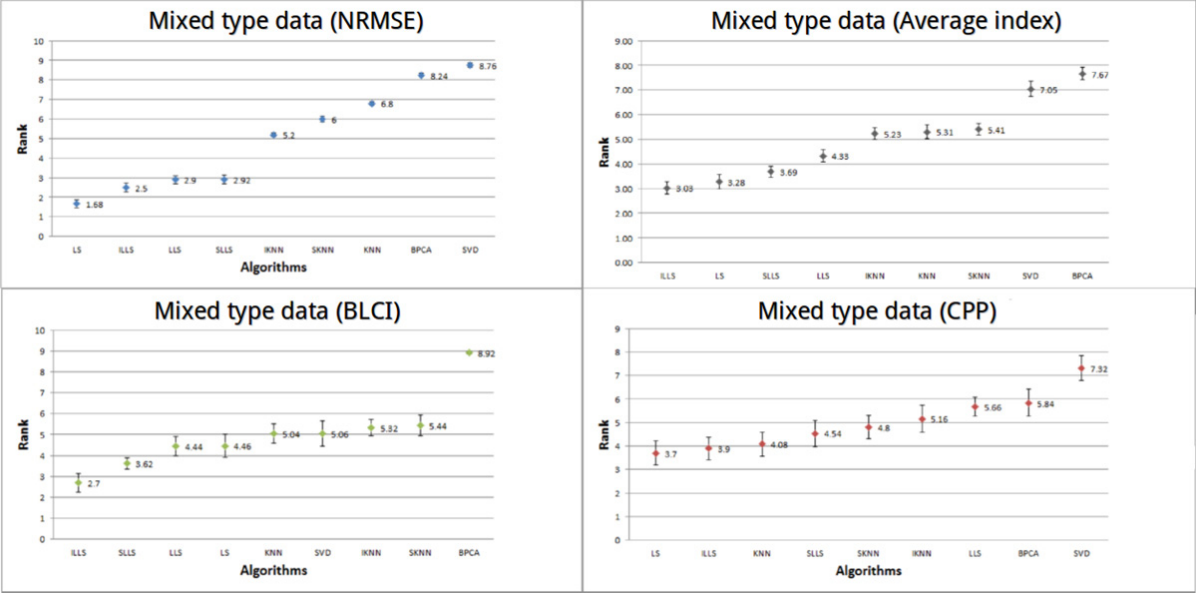
**Performances of different methods on mixed type datasets**. In the mixed type datasets, the performances of all algorithms were estimated by three indices (NRMSE, CPP and BLCI) and the average index. Each point represents the average rank for each algorithm. Different colors (blue, red, green and gray) represent the results evaluated by different indices. The error bar is the standard error.

In Figure 6, we can obviously see that LS has a low average rank (1.68) using NRMSE. However, the performance of LLS-like algorithms is better than that of LS using BLCI. Using CPP, the average rank of LS is 3.7, the average rank of ILLS is 3.9, the average rank of KNN is 4.08 and the average rank of SLLS is 4.54. The top three performing algorithms (ILLS, LS and SLLS) are all very competitive with each other. The top performing algorithm is ILLS, followed by LS and SLLS.

#### Performance of imputation algorithms on all data

**Figure 7:**
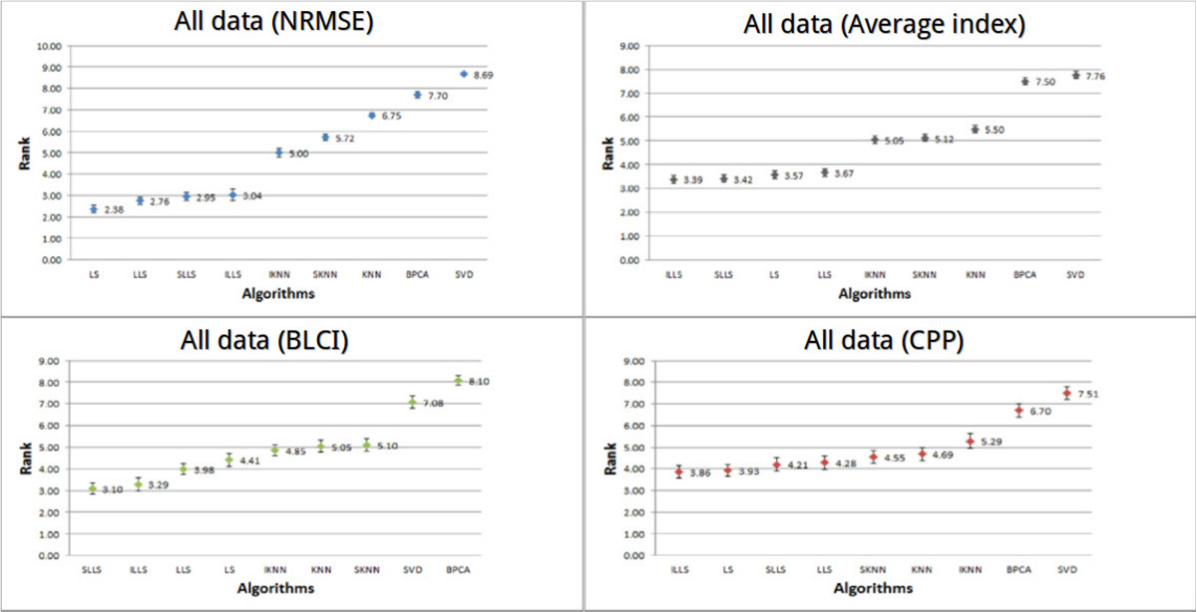
**Performances of different methods on all datasets**. In all datasets (thirteen datasets), the performances of all algorithms were estimated by three indices (NRMSE, CPP and BLCI) and the average index. Each point represents the average rank for each algorithm. Different colors (blue, red, green and gray) represent the results evaluated by different indices. The error bar is the standard error.

Performance of each algorithm using the three kinds of indices and the average index on all datasets is given in Figure 7. It can be clearly seen that the performances of LLS-like algorithms and LS are better than the performances of KNN-like algorithms. We noted that no algorithm can perform well on all kinds of datasets. Therefore, the best algorithm cannot be found, but we can find the optimal algorithm for each data type (shown in Table 3).

**Table 3:** The optimal algorithm determined by using various indices for different types of datasets.

Index	Data	Best algorithm
*NRMSE*	Time series	ILLS
	Non-time series	LS
	Mixed type	LS
	All Data	LS

*CPP*	Time series	ILLS, LLS
	Non-time series	SKNN
	Mixed type	LS
	All data	ILLS

*BLCI*	Time series	SLLS
	Non-time series	SKNN
	Mixed type	ILLS
	All data	SLLS

*Average index*	Time series	SLLS
	Non-time series	LS
	Mixed type	ILLS
	All data	ILLS

### Robustness of each imputation algorithm

Tuikkala *et al. *demonstrated that BPCA is the best imputation method on most of datasets [33], while *de *Brevern *et al. *indicated that KNN constitutes one efficient method for restoring the missing values with a low error level [10]. According to our experiences, BPCA does not always perform well on all benchmark datasets, and the performance of KNN is usually worse than that of other methods for most of time, which means that KNN cannot accurately estimate missing values to improve downstream analysis. Integrating the results of the previous studies with our experiences, it strongly suggests that the optimal imputation algorithms for different types of datasets may be different. Therefore, it is necessary to compare the robustness of each imputation method, which is useful for choosing an optimal algorithm for most of the researchers, especially when they cannot ensure the type of their dataset.

#### Robustness against different data types

**Figure 8:**
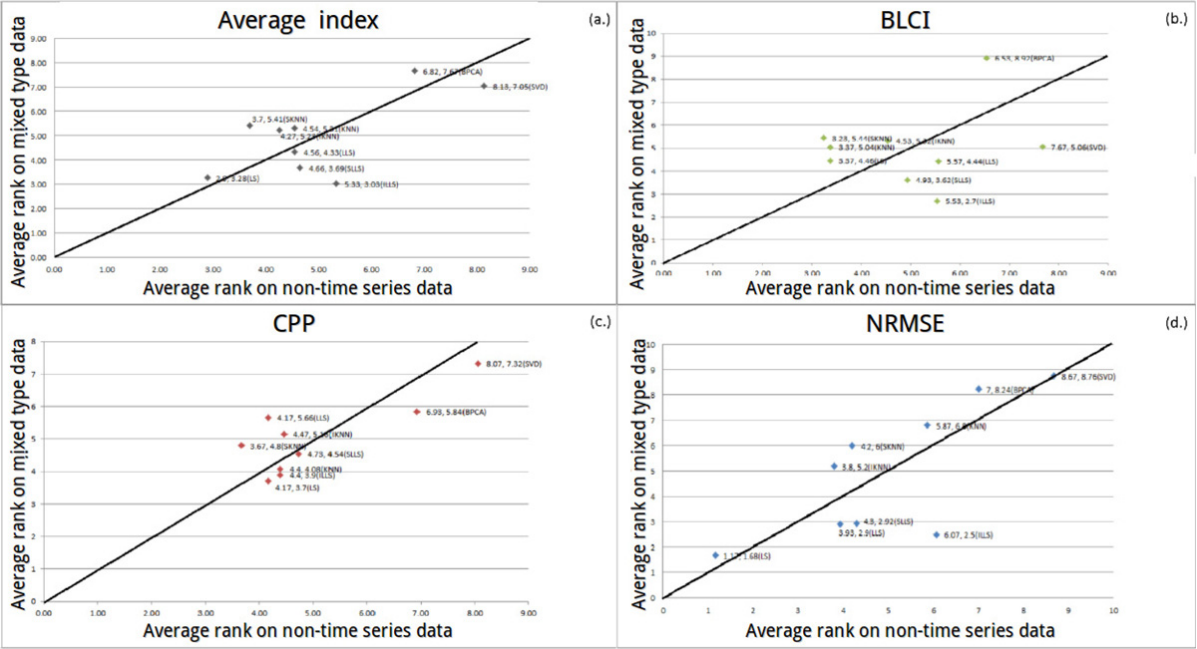
**The coordinate of a point means that the average rank of each algorithm on two types of datasets (non-time series and mixed type)**.

LS outperforms other algorithms using NRMSE (in Figure 8d) and the average index (in Figure 8a). In Figure 8a and 8d, ILLS and SKNN are more sensitive than the other algorithms. When illustration has no explicit trend, we set a threshold *σ *(*σ *= |(non-time series average rank) - (mixed type average rank)|). When *σ *is less than 1.5, it indicates that the performance of an algorithm is not much different between datasets. In Figure 8c, the performance is not much different between LLS-like algorithms and KNN-like algorithms in mixed type dataset against non-time series dataset. In Figure 8b, LS, LLS, IKNN, KNN and SLLS are also not much different. On the other hand, ILLS, SKNN, BPCA and SVD are sensitive algorithms. Therefore, in Figure 8b and 8c, we suggest that LS can be used when researchers cannot ensure the type of their dataset.

**Figure 9:**
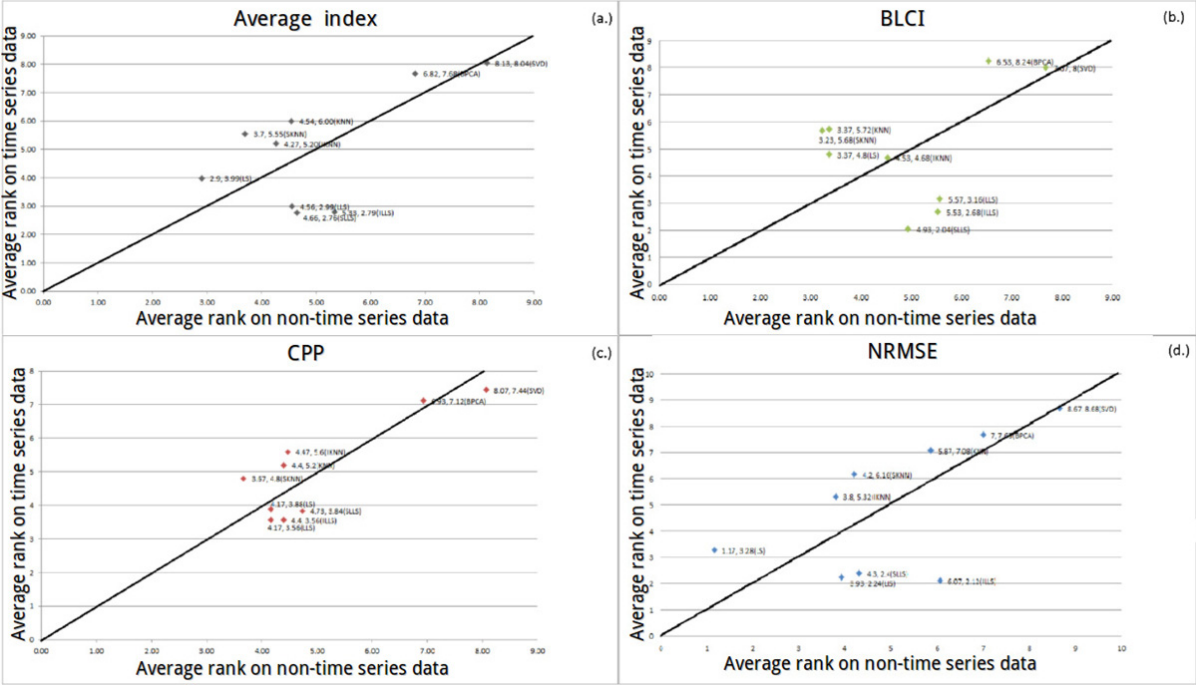
**The coordinate of a point means that the average rank of each algorithm on two types of datasets (non-time series and time series)**.

**Figure 10:**
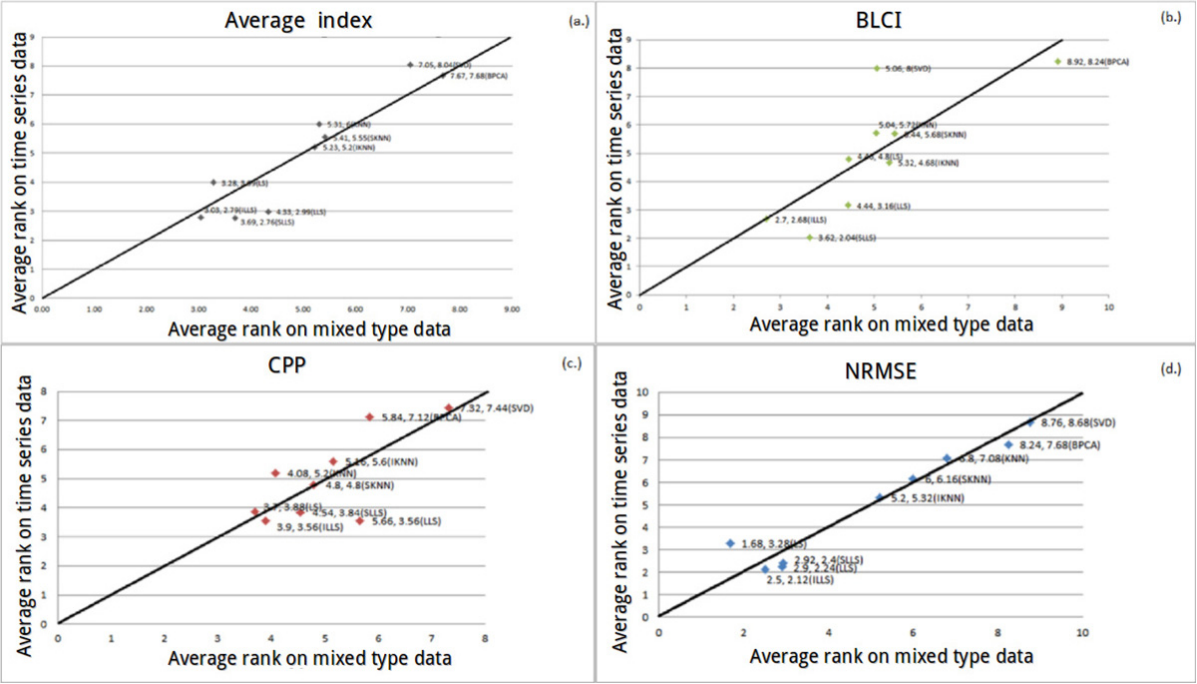
**The coordinate of a point means that the average rank of each algorithm on two types of datasets (mixed type and time series dataset)**.

There is an obvious trend in Figure 9a and 9d. Hence, we recommend that LS can be used when researchers cannot ensure whether their dataset belongs to time series dataset or non-time series dataset. In Figure 9c, LS is the optimal algorithm (*σ *is less than 1.5 and the algorithm is close to left-down) when researchers cannot ensure the type of their datasets. In Figure 9b, LS is still the best one when the type of the dataset is unknown. In Figure 9d, it can be obviously seen that ILLS and LS are more sensitive than the other algorithms. In Figure 9a, LLS-like algorithms prefer time series datasets but not non-time series datasets. SKNN prefer non-time series datasets but not time series datasets. In Figure 9b, ILLS, SLLS and LLS prefer time series datasets but not non-time series datasets. KNN and SKNN prefer non-time series datasets but not time series datasets. In Figure 10c, LLS is more sensitive than the other algorithms. In Figure 10b, SVD prefers mixed type datasets but not time series datasets. In Figure 10a and 10b, ILLS is considered as the optimal algorithm, which can be used when the type of the dataset is either time series or mixed type. In Figure 10d, the performances of all algorithms are similar between LS and LLS-like algorithms, but LS is still more sensitive than other algorithms. In Figure 10c, ILLS and LS have better performances than the other algorithms.

#### Robustness against data from different species

**Figure 11:**
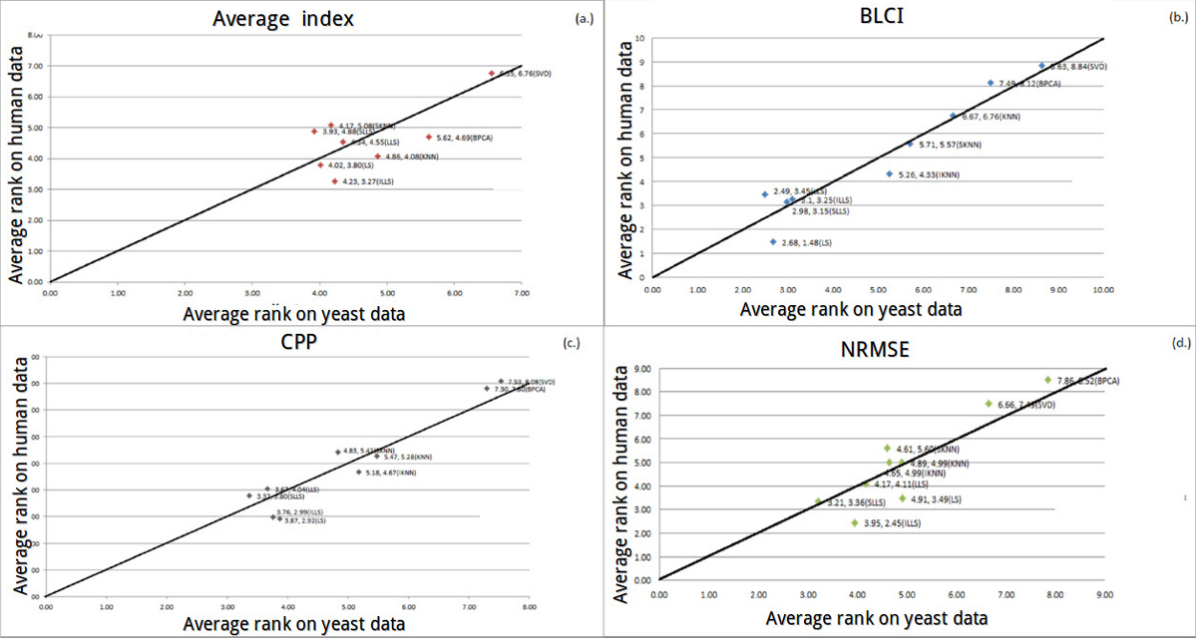
**The coordinate of a point means that the average rank of each algorithm on two organisms (yeast and human)**.

From Figure 11a to 11d, we can see that *σ *is almost less than 1 for each point (*σ *= |Human average rank - Yeast average rank|). This indicates that the performance of each algorithm between different organisms is very similar.

### An easy-to-use web tool for missing value imputation

**Figure 12:**
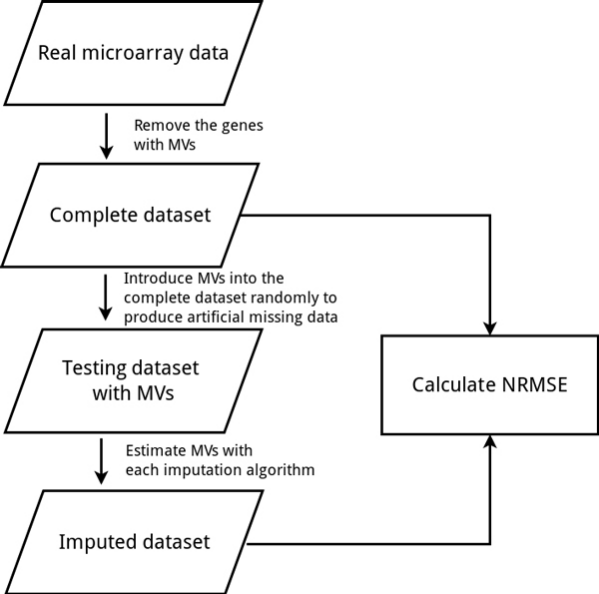
**The workflow of performance comparison in MissVIA**.

**Figure 13:**
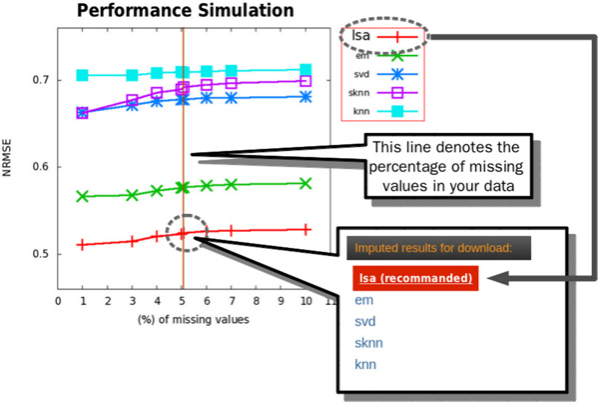
The plot of various missing rates vs. NRMSE generated by MissVIA through the procedure of performance comparison.

In addition to a comprehensive comparison between imputation algorithms, we developed a web-based imputation tool--MissVIA to help researchers, who do not have good programming skills, to deal with missing values in their datasets. In MissVIA, many existing imputation algorithms were integrated together. MissVIA is built up based on the easy-to-use principle, so every imputation task could be completed with only three steps: (a) upload the dataset with missing values, (b) choose the imputation algorihtms and (c) click the "Submit" button. Once MissVIA receives the request of an imputation task, it will send an e-mail notice with the link of the job to users. Subsequently, MissVIA will initiate a simulation procedure for performance comparison to find out the optimal algorithm (see Figure 12). Finally, the results of performance comparison would be presented with a missing rate-to-NRMSE plot (see Figure 13). According to the plot, MissVIA would determine the optimal algorithm, and then users can use the imputed result for the downstream analysis.

## Conclusions

To find an optimal method to solve the missing value problem efficiently, we conducted a comprehensive performance comparison of various missing value imputation algorithms in this work. First, we investigated the impact of different types of microarray data on the performance of imputation methods. Three types of microarray data (time series, non-time series and mixed type) were used as benchmark datasets, and the performance of each algorithm was evaluated using three kinds of measures (NRMSE, CPP and BLCI) and the average of these measures (called the average index). These measures are originally used for different purposes. NRMSE is for estimation of deviation between the estimated values and the real values, CPP is for evaluation of clustering results, and BLCI is for assessing the results of finding differentially expressed genes. Our results suggest that, for time series data, ILLS and SLLS have better performances if one wants to do clustering analysis or find differentially expressed genes. For non-time series data, LS is the best algorithm when the performance is evaluated using NRMSE, while SKNN is better than the others if one wants to conduct downstream microarray data analysis. For mixed type data, ILLS is the best choice if one wants to find differentially expressed genes, but LS would be better for the other two purposes.

Then we investigated whether the microarray data from different species would affect the performance of various imputation methods or not. Our results indicate that what kind of species a dataset comes from does not have any obvious effect on the performance of imputation methods. This means that when one is dealing with missing values, what he needs to consider is not the species that the dataset comes from, but the type of the dataset. Besides, we used a distinct illustration to display the relationship between different types of datasets, which is helpful to reveal the robustness of these imputation methods and is useful for researchers to choose an optimal algorithm for their datasets. Besides, to assist experiment practioners in solving missing value problems directly before data analysis, we developed a web-based imputation tool. In this web tool, only 3 steps are needed, and then users could easily obtain a complete dataset imputed by the optimal algorithm.

## Competing interests

The authors declare that they have no competing interests.

## Authors' contributions

WSW conceived the research topic and provided essential guidance. CCC developed the web-based tool, and he did all the simulations with SYC. CCC, SYC, and WSW wrote the manuscript. CCW helps to revise the manuscript. All authors have read and approved the final manuscript.

## Declarations

The full funding for the publication fee came from Taiwan National Science Council and College of Electrical Engineering and Computer Science, National Cheng Kung University.

This article has been published as part of *BMC Systems Biology *Volume 7 Supplement 6, 2013: Selected articles from the 24th International Conference on Genome Informatics (GIW2013). The full contents of the supplement are available online at http://www.biomedcentral.com/bmcsystbiol/supplements/7/S6.
